# Immunoinformatics and Structural Analysis for Identification of Immunodominant Epitopes in SARS-CoV-2 as Potential Vaccine Targets

**DOI:** 10.3390/vaccines8020290

**Published:** 2020-06-09

**Authors:** Sumit Mukherjee, Dmitry Tworowski, Rajesh Detroja, Sunanda Biswas Mukherjee, Milana Frenkel-Morgenstern

**Affiliations:** Cancer Genomics and BioComputing of Complex Diseases Lab, Azrieli Faculty of Medicine, Bar-Ilan University, Safed 1311502, Israel; sumit.mukherjee@biu.ac.il (S.M.); dmitwor@gmail.com (D.T.); rajesh.detroja@biu.ac.il (R.D.); mukhers1@biu.ac.il (S.B.M.)

**Keywords:** SARS-CoV-2, COVID-19, immunodominant epitope, HLA, vaccine target

## Abstract

A new coronavirus infection, COVID-19, has recently emerged, and has caused a global pandemic along with an international public health emergency. Currently, no licensed vaccines are available for COVID-19. The identification of immunodominant epitopes for both B- and T-cells that induce protective responses in the host is crucial for effective vaccine design. Computational prediction of potential epitopes might significantly reduce the time required to screen peptide libraries as part of emergent vaccine design. In our present study, we used an extensive immunoinformatics-based approach to predict conserved immunodominant epitopes from the proteome of SARS-CoV-2. Regions from SARS-CoV-2 protein sequences were defined as immunodominant, based on the following three criteria regarding B- and T-cell epitopes: (i) they were both mapped, (ii) they predicted protective antigens, and (iii) they were completely identical to experimentally validated epitopes of SARS-CoV. Further, structural and molecular docking analyses were performed in order to understand the binding interactions of the identified immunodominant epitopes with human major histocompatibility complexes (MHC). Our study provides a set of potential immunodominant epitopes that could enable the generation of both antibody- and cell-mediated immunity. This could contribute to developing peptide vaccine-based adaptive immunotherapy against SARS-CoV-2 infections and prevent future pandemic outbreaks.

## 1. Introduction

A new coronavirus, severe acute respiratory syndrome coronavirus 2 (SARS-CoV-2), has recently emerged as a human pathogen that causes fever, pulmonary disease, and pneumonia [1,2,3]. Following an outbreak that initiated in China, human-to-human infection has spread rapidly across the world. The COVID-19 global pandemic is more severe than previous coronavirus-related outbreaks caused by severe acute respiratory syndrome coronavirus (SARS-CoV) and Middle-East respiratory syndrome coronavirus (MERS-CoV) [4,5,6]. By 30 May 2020, over 6,066,500 people were infected and 367,500 people had died globally from COVID-19. No licensed vaccine is presently available for this disease, although several vaccines are in initial clinical trial stages [7]. Given the magnitude of this international public health emergency, universal vaccines are urgently needed to control the COVID-19 pandemic. 

In the postgenomic era, the availability of vast sequence data from pathogens and the advancement of computational prediction tools have greatly facilitated identifying potential immunogenic epitopes in pathogen proteins. This can be useful in designing vaccines against designated pathogens [8,9]. In recent studies, promising vaccine candidates were predicted from viral pathogens based on immunoinformatics approaches [10,11,12,13,14,15,16]. The computational prediction of novel vaccine targets and immune responses against complex pathogenic diseases have minimized the time needed for in-vitro experiments in developing vaccines [17]. The spread of COVID-19 is a global crisis and emergency at this time. Sufficient information is not yet available regarding the components of the SARS-CoV-2 sequence that are recognized by human immune responses. Clearly, the identification of immunogenic regions from the SARS-CoV-2 sequence and the prediction of immune responses are crucial for designing vaccines against SARS-CoV-2. 

In the present research, we used an immunoinformatics-based computational approach to mine the proteome of SARS-CoV-2 and subsequently identify immunodominant epitopes of SARS-CoV-2. Detecting immune responses that are based on specific immunodominant epitopes enables generating both antibody- and cell-mediated immunity against a specific pathogen. This can facilitate the fast and effective elimination of the pathogen. Using a variety of computational tools, we predicted all possible B- and T-cell epitope regions in SARS-CoV-2 protein sequences, and selected those regions where both B- and T-cell epitopes mapped. Notably, despite evidence that SARS-CoV-2 originated from bats, it phylogenetically diverged from SARS-CoV [18,19]. Therefore, we selected and mapped regions that positively predicted protective antigens to the experimentally validated epitopes of SARS-CoV. We then only selected epitopes that were 100% identical between SARS-CoV and SARS-CoV-2. We thus identified 15 potential immunogenic regions and 25 immunodominant epitopes from SARS-CoV-2 proteins. The global distribution of these epitopes was analyzed to estimate the percentage of infected individuals that express an MHC molecule that is capable of binding a particular epitope. Interestingly, seven epitopes were found to cover more than 87% of the worldwide virus-affected population. Further structural molecular docking analyses were carried out to estimate the binding interactions of these potential epitopes with human major histocompatibility complexes (MHC) proteins. Thus, our study has identified a set of potential immunodominant epitopes from the SARS-CoV-2 proteome that are capable of generating both antibody- and cell-mediated immune responses. To conclude, our findings may be useful in developing effective peptide vaccines against COVID-19 infections.

## 2. Materials and Methods 

### 2.1. Immunoinformatics Analysis

#### 2.1.1. Data Retrieval

We retrieved the whole genome and proteome of SARS-CoV-2 isolates from different geographic locations from Genbank (NCBI). Protein sequences of SARS-CoV and MERS-CoV were also collected from Genbank. The experimentally determined B- and T-cell epitopes of SARS-CoV were retrieved from the publicly available Immune Epitope Database (IEDB) [20] with the filtering criteria of at least one positive assay: (i) positive B-cell assays, (ii) positive T-cell assays, and (iii) positive MHC binding assays.

#### 2.1.2. Predicting Potential Linear B-cell Epitopes in SARS-CoV-2

Linear B-cell epitopes are peptides with antigenic abilities that are bound by receptors on the surface of B lymphocytes and, thus, generate immune responses [21]. We used multiple approaches to predict the linear B-cell epitopes from the protein sequences of SARS-CoV-2. These included three machine learning-based methods, namely, BepiPred [22], ABCpred [23], and LBtope [24]. BepiPred utilizes data that are obtained from three-dimensional (3D)-structures of the antigen-antibody complex, based on random forests that were trained on the B-cell epitope. We set a cutoff of 0.5 for detecting B-cell epitopes using BepiPred. The ABCpred and LBtope methods are based on artificial neural networks trained on similar B-cell epitope positive data. ABCpred relies on random peptides for the training of negative data, in contrast to LBtope, which uses negative data that are based on experimentally validated non-B-cell epitopes from IEDB [20]. We used a cutoff of 0.51 and chose all window lengths of 10–20 for predicting B-cell epitopes using the ABCpred search tool.

#### 2.1.3. Prediction of Potential T-cell Epitopes in SARS-CoV-2

Predicting T-cell epitopes is important for identifying the smallest peptide in an antigen that is able to stimulate CD4 or CD8 T-cells to generate immunogenicity. Thus, the aim here is to identify peptides within antigens that are potentially immunogenic. MHC-peptide binding is considered to be the most important determinant of T-cell epitopes [25]. MHC binds to the antigenic region and becomes more available on the cell surface, where T-cells can recognize them. The accurate prediction of these binders is crucial for efficient vaccine design due to the importance of MHC binders for the activation of T-cells of the immune system [26]. MHC class I and II epitopes were predicted using Tepitool [27], available at IEDB [28]. For predicting MHC class-I epitopes, the parameter for selecting predicted peptides was set as equal or less than 500 median inhibitory concentrations (IC50), while for MHC class-II epitope prediction, the same parameter was set to equal or less than 1000 nM IC50 [29,30]. NetMHCpan-4.0 [31] and nHLAPred [32] were also used to predict the MHC class-I binding epitope, and potential T-cell epitopes were predictedusing CTLPred [33]. CTLPred predicts T-cell epitopes (CTL) from antigen sequences instead of using the intermediate step in which MHC Class I binders are predicted. 

#### 2.1.4. Prediction of Protective Antigens

It is important to identify epitopes that are crucial for inducing protection and eliminate others in order to develop peptide-based vaccines. Protective antigens are able to induce an immune response. Thus, Vaxijen V2.0 [34] was used to predict the ability of the predicted SARS-CoV-2 epitopes to protect antigens. The default threshold of Vaxijen V2.0 (0.4) was used to predict the protection potential of antigens. 

#### 2.1.5. Analysis of Epitope Conservation and Population Coverage of T-cell Epitopes

An IEDB conservancy analysis tool was utilized in order to analyze the degree of conservation of SARS-CoV-2 B- and T-cell epitopes. The population coverage of T-cell epitopes was analyzed using tools available at the IEDB [20]. The predicted population coverage represents the percentage of individuals within a defined population which are likely to elicit an immune response to a T-cell epitope.

#### 2.1.6. Prediction of Allergenicity, Toxicity and Possibilities of Autoimmune Reactions

The allergenicity of immunodominant epitopes were predicted using AllerTOP v. 2.0 [35] and AlgPred [36]. AllerTOP v. 2.0 classified allergens and non-allergens based on the k-nearest neighbours (kNN) method within an accuracy of 88.7%. AlgPred classified allergens and non-allergens using a hybrid approach (SVMc, IgE epitope, ARPs BLAST, and MAST) within an accuracy of 85%. The toxicity of the epitopes was predicted by means of the ToxinPred [37] web-server, which applies machine learning approaches using different properties of the peptides. Further, we performed a BLAST search (with a criteria of >90% identity) [38] of all of the potential epitopes vs. all the available human antigens from positive B-cell/T-cell/MHC ligand assays for autoimmune diseases in IEDB to determine the risks of potential predicted epitopes triggering a cascade of autoimmune reactions.

### 2.2. Structural Analysis

#### 2.2.1. Data Collection for Structural Analysis

Peptide epitopes of various lengths (ranging from 7 to 20 residues) which presented on MHC Class I and II molecules were retrieved from SCEptRe (Structural Complexes of Epitope Receptors) [39], AutoPeptiDB [40], and Protein Data Bank (PDB) [41].

#### 2.2.2. Modeling of Epitope MHC-bound Conformations 

Backbone conformations of the peptides that were bound to human leukocyte antigen (HLA) proteins were collected from PDB, clustered, and used as structural templates for 3D modeling of the epitopes (identified as immunodominant in the immunoinformatics study). Based on similarities and common structural patterns in HLA-peptide binary complexes, we generated 3D structures of the epitopes that are listed in Table 1, in their bound conformations. The confrontations of peptide side-chains were built using SCWRL [42]. 

#### 2.2.3. Molecular Docking

Docking grids were generated by the autogrid module of AutoDock4 application [43], using the default values of the van der Waals scaling factor (0.8) and charge cutoff (0.15). A cubic box 35 Å in length was centered on the ligand in the active site of each protein structure. The OpenBabel modules [44] and Chimera v1.11.2 [45] were used to prepare peptides and target HLA proteins for docking. Ionization states were calculated at pH 7.0 ± 2.0. The conformers of peptide molecules were generated and docked to the protein using AutoDock4 and AutoDock Vina [46]. The molecules were docked to the canonical Site1 binding region, and docking conformations with the best scores were analyzed. The epitopes with the most promising characteristics were selected for further analysis and optimization. These characteristics included favorable interactions and top-ranked AutoDock Vina scores, together with acceptable conformations, consistent with peptide recognition by MHC Class I and II structural frameworks.

## 3. Results

### 3.1. Identification of Immunodominant Epitopes from the Proteins of SARS-CoV-2

**Figure 1:**
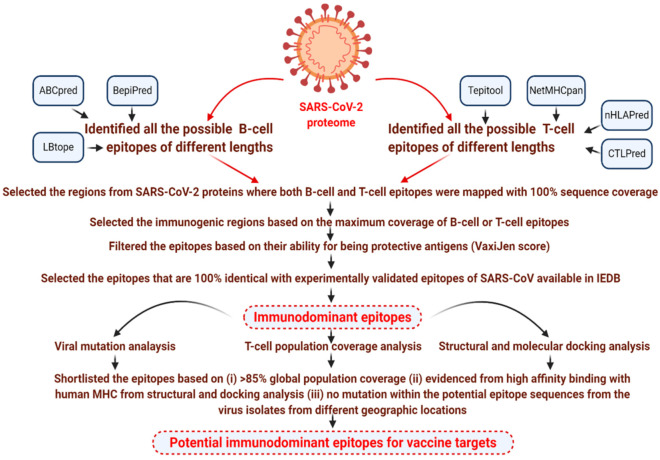
Computational approaches used to identify potential immunodominant epitopes from the proteome of severe acute respiratory syndrome coronavirus 2 (SARS-CoV-2).

Immunodominant epitopes, which can generate both antibody- and cell-mediated immunity, were identified to generate memory cells against SARS-CoV-2. We first predicted B- and T-cell epitopes and their possible MHC alleles from the SARS-CoV-2 protein using a variety of tools described in the Methods in order determine immunodominant epitopes (Section 2.1.2 and Section 2.1.3). All of the B- and T-cell epitopes that were predicted from the different SARS-CoV-2 protein sequences were selected for further analysis. Subsequently, using a combinatorial screening approach, we analyzed all of the predicted B-cell and T-cell epitope (MHC-I and MHC-II) libraries of different lengths, from all protein sequences. The aim was to identify the immunogenic regions that could potentially act as both B-cell and T-cell epitopes. We compared the libraries of predicted B-cell epitopes *vs.* T-cell epitopes and selected those epitopes with 100% sequence coverage. The lengths of the immunogenic regions were selected based on the maximum coverage of B-cell or T-cell epitopes in the mapped regions. Figure 1 depicts the pipeline used in the study for detecting immunodominant epitopes. 

**Figure 2:**
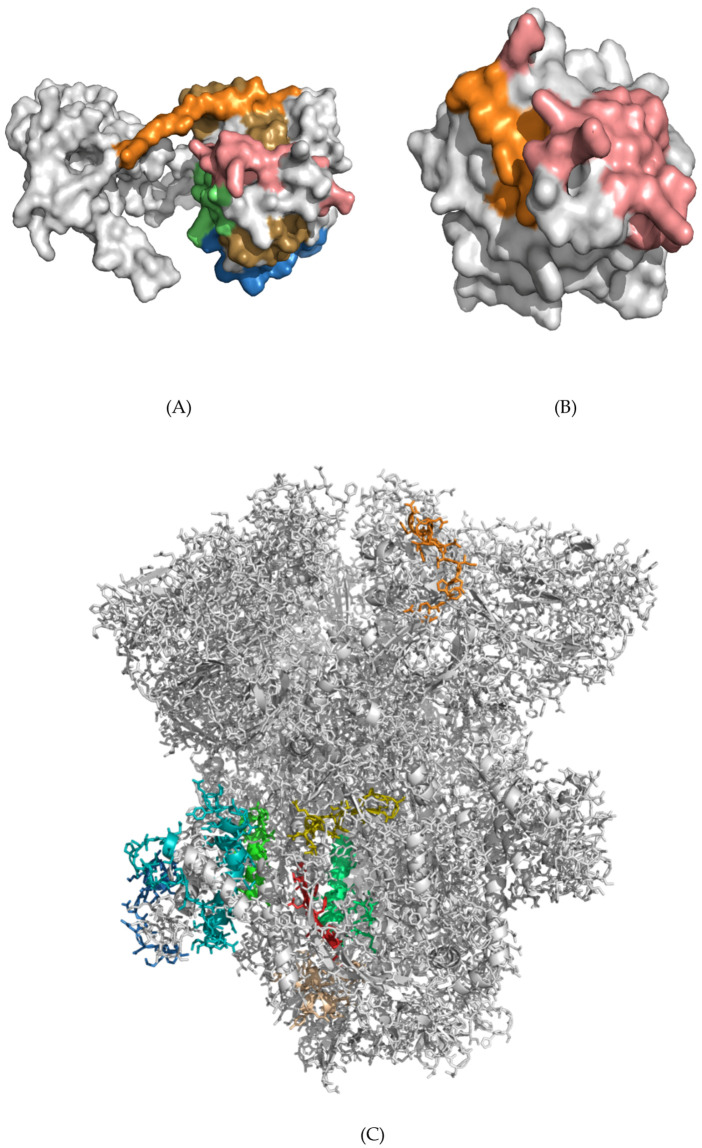

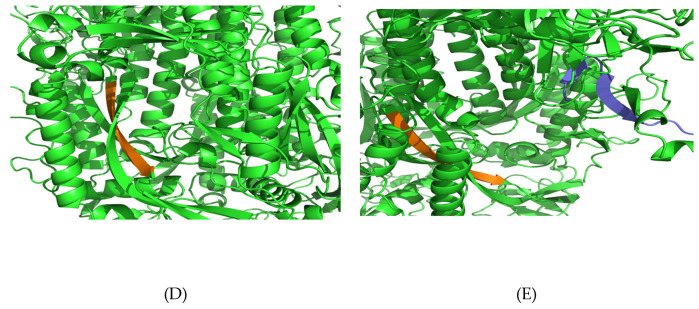
Potential immunogenic regions mapped onto SARS CoV-2 proteins. **(A)** Nucleocapsid phosphoprotein: residues 176–191 (orange), 240–264 (salmon), 268–286 (lime), 292–330 (sand), 360–375 (sky-blue). **(B)** Membrane glycoprotein: residues 61–70 (orange), 157–187 (salmon). **(C)** Spike glycoprotein: residues 327–343 (orange), 663–680 (yellow), 817–833 (wheat), 891–918 (green), 1019–1041 (lime), 1060–1068 (red), 1157–1209 (cyan), and 1254–1273 (sky-blue). In all of these regions, both B-cell and T-cell epitopes are mapped. **(D)** The region Val1060–Val1068 (orange beta-strand) of the spike glycoprotein (green cartoon) is mostly composed of hydrophobic residues (VVFLHVTYV) which are not exposed to solvent. **(E)** Residues Asp663–Leu680 (DIPIGAGICASYHTVSLL, blue) of the spike glycoprotein (green cartoon) are mostly solvent-exposed, with the exception of Cys671 and Ala672 (Table S2).

We predicted the abilities of the epitopes to serve as protective antigens using Vaxijen and to understand the immunomodulatory effect of epitopes identified from immunogenic regions [34]. Unique epitopes were selected accordingly for further analysis. We identified a total of 17 immunogenic regions from the viral membrane glycoprotein, spike glycoprotein, and nucleocapsid phosphoprotein, onto which both B-cell and T-cell epitopes were mapped. Although immunoinformatics approaches were established to identify potential epitopes from pathogens, some computationally predicted epitopes may not be optimally immunogenic *in vivo*. Therefore, it is necessary to test the predicted epitopes *in vivo* to ensure that they can generate B-cell and/or T-cell responses. Detailed understanding of protective immune responses against SARS-CoV might be presumably important for developing a vaccine against SARS-CoV-2 [47]. For this reason, the 100% identical and experimentally confirmed epitopes between SARS-CoV and SARS-CoV-2 were chosen in this study. Accordingly, we mapped all of the epitopes that were predicted from the 17 regions of three proteins of SARS-CoV-2 with the experimentally validated epitopes of SARS-CoV, and only selected the 100% identical epitopes. The lengths of the epitopes were adjusted based on the mapped experimentally-determined epitopes of SARS-CoV. To define the immunodominant epitopes, the core parts of both B-cell and T-cell epitopes were verified within those mapped epitope sequences. Finally, we found 15 potential immunogenic regions of SARS-CoV-2 that explicitly include 25 mapped immunodominant epitopes, which can generate immune responses by both B-cells and T-cells (Table 1, Figure 2A–C, and Table S1).

Interestingly, the mapping of immunogenic regions onto the structure of SARS-CoV-2 spike glycoprotein (Figure 2C) revealed a number of potential epitopes that were not exposed to solvent (Tables S2 and S3). For example, the beta-strand spanning Val1060–Val1068, composed of hydrophobic residues (VVFLHVTYV), is not a solvent-accessible region in the multi-subunit spike glycoprotein (Figure 2D). Indeed, the solvent-accessible surface area (SASA) was estimated to be ~0 for all residues of this epitope, with the only exception of theVal1068 (SASA ~24 A^2^, Table S2). This region contrasts with the nearby region of another epitope, Asp663–Leu680 (DIPIGAGICASYHTVSLL, Table 1), which was mostly exposed to solvent (Figure 2E, Table S2). This implies the “recognition-after-proteolysis” pathway of protein interactions with the immune system.

### 3.2. Analysis of Viral Mutations within the Potential Epitope Regions

**Figure 3:**
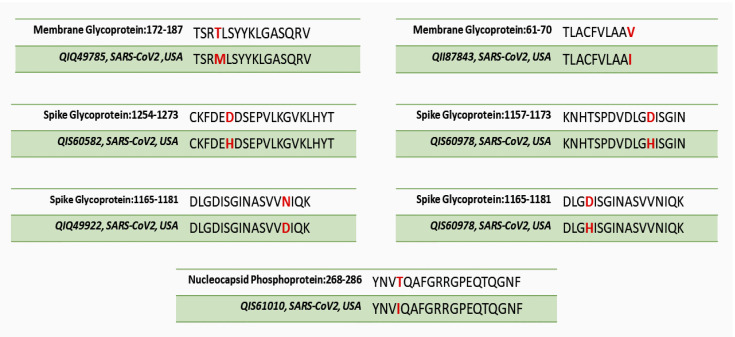
The point mutations found within the potential epitopes of American SARS-CoV-2 isolates. The mutated regions are highlighted in red.

Selection pressure of the human immune system has been shown to drive viral point mutations that evade immune surveillance [48]. Therefore, patterns of mutational events need to be examined in order to understand the epitope escape that is important for the transmission of viruses between different sub-populations. Potential immunogenic epitopes with a low chance of mutation are thus optimal candidates for generating effective vaccines. We analyzed mutations within the immunodominant epitopes identified in SARV-CoV-2 isolates from different geographic locations. We found a few single point mutations within the immunodominant regions of a few SARS-CoV-2 sequences isolates from the USA (Figure 3). Despite the low number of point mutations in the immunodominant epitopes, they reflect the severity of mutated viral genomes within the American population. Our observations highlight that immune pressure-induced genetic drifts play an important role in the evolution of SARS-CoV-2. This might be essential for evading immune surveillance by the host. The correlation of patterns of mutations and human immune pressure-induced genetic evolution of SARS-CoV-2 will be understood in detail with the availability of more sequenced viruses from different countries. 

### 3.3. Population Coverage of Immunodominant Epitopes

Human leukocyte antigens (HLAs) are the most polymorphic genes in humans, and their allele distributio and expression vary by ethnic group and geographical location. The classical HLA loci are class I (HLA-A, B, C, E, F and G) and class II (HLA-DR, DQ, DM and DP) molecules, which provide antigen presentation to CD8 and CD4 T-cells [49]. Therefore, the identification of epitopes that can be recognized by multiple HLA alleles and cover most of the world’s population is important for the development of successful vaccines. Thus, we analyzed population coverage by HLAs of all of the epitopes from the immunogenic regions of SARS-CoV-2 using the IEDB population coverage analysis tool [20]. We identified seven epitopes from five immunogenic regions, which cover more than 87% of the world’s population (Table 2). Among these seven potential immunodominant epitopes, six are 17 amino acids in length. We found that the residue 891–918 region of the spike glycoprotein contains three potential immunodominant epitopes. Of these, two have world population coverages of 97.46% and 92.52%, respectively. Similarly, the residue 292–330 region of the nucleocapsid phosphoprotein contains three potential immunodominant epitopes. Of these, two have 87.42% and 92.81% world population coverages, respectively. These results indicate that the seven immunodominant epitopes could be potential candidates for designing vaccines against SARS-CoV-2 that can cover almost the entire world population.

### 3.4. Analysis of Allergenicity, Toxicity and Autoimmune Reactivity

Epitope allergenicity is a prominent obstacle for vaccine development. We thus verified that the identified epitopes are not allergens. The allergenicity analysis results of the seven immunodominant epitopes (Table 2) highlighted that six of these epitopes were not predicted as allergens using bothAllerTOP [35] and AlgPred [36]. Only one epitope (“FIEDLLFNKVTLADAGF”) was predicted as an allergen by AllerTOP, whereas the AlgPred method predicted it as a non-allergen. Therefore, the proper classification of allergens was not possible for this epitope due to the limitation of computational prediction methods. Toxicity profiling of these predicted epitopes revealed that all were safe and possibly non-toxic. Epitope spreading is a process where diversification of the immune response is induced by an antigen to meet both B-cell and T-cell specificities during a chronic autoimmune or infectious response [50,51]. Thus, we analyzed the possibility that the seven predicted immunodominant epitopes (Table 2) would generate autoimmune reactions. For this purpose, we performed a BLAST search of our epitopes against the database of epitope sequences of human antigens for autoimmune diseases, which were validated by positive B-cell/T-cell/MHC ligand assays. Consequently, we found that none of the human epitopes for autoimmune disease share significant sequence identity with our predicted SARS-CoV-2 immunodominant epitopes (Table 2). This result indicates that the seven epitopes have a very low risk for generating autoimmune reactions in humans.

### 3.5. Structural Analysis and Modeling of Epitope Presentation by MHC Class I and II Systems

Epitopes are faced with extremely complex and competitive environments that include the multitude of HLA proteins that bind immunogenic peptides with different affinities, and present selected epitopes to surface receptors on immune cells. Therefore, we performed molecular docking analysis to understand the binding interactions of the identified immunodominant epitopes with human MHC complexes. 

**Figure 4:**
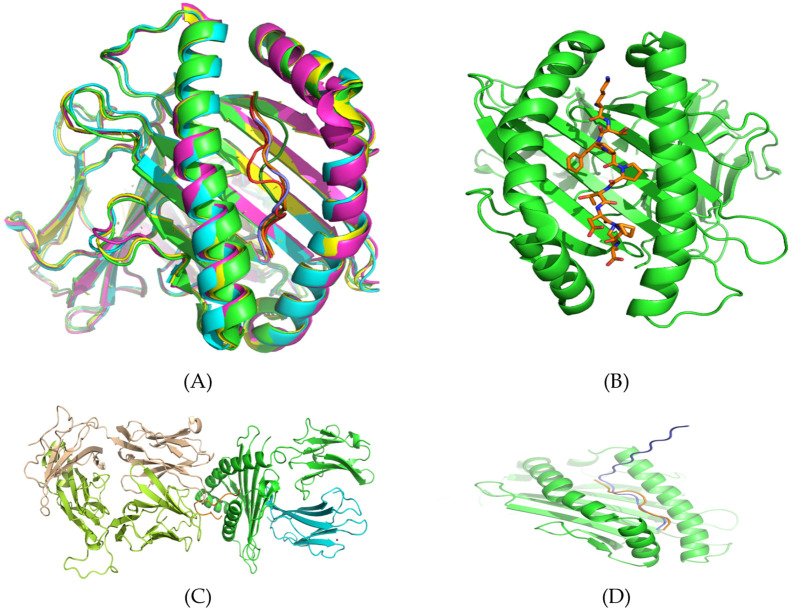
(**A**) Structures of aligned major histocompatibility complexes (MHC) (Class I) HLA-peptide complexes are fairly similar, sharing the same canonical fold and peptide binding mode. (**B**) A typical 9-mer peptide molecule (orange sticks) arranged in the binding site of a HLA protein (green cartoon) from MHC Class I. The N- (upper side) and C- (down side) termini occupy essentially the same positions in the A- and F-pockets, respectively (**C**) A typical 13-mer peptide in “bulged” conformation (orange chain) confined to the interface area between its cognate HLA-B*35:08 protein (alpha chain, green; beta-micro-globulin, blue) and the T-cell receptor (alpha chain, wheat; beta-chain, lemon). PDB ID: 2ak4 (**D**) The elongated 17-residue epitope (blue chain, LNKHIDAY**KTFPPTEPK**) bound to HLA from MHC Class I (green ribbon), with the N-terminal part arranged outside the A-pocket. The C-terminal part is bound in the F-pocket of the binding groove. The binding mode of the core epitope **KTFPPTEPK** (orange chain) is also shown.

Structures of different HLA-peptide complexes from MHC class I and II were collected and aligned, as described in Methods. Structures of HLAs are fairly similar within each group (I and II) and share the same canonical fold. The epitopes were clustered in similar conformations in the HLA antigen binding grooves created by two helices in parallel orientation (Figure 4A,B). For the most part, backbone “traces” of peptides were similar (Figure 4A). The N- and C-termini occupied essentially the same positions inpockets A and F of HLA binding sites (Figure 4C,D). This suggests that conformational flexibility was mostly concentrated in the middle part of the epitope sequences, whereas the motion of terminal residues was restricted, in agreement with the possibility of “bulged” conformations. Based on these similarities and common canonical structural properties in HLA-peptide binary complexes, we generated 3D structures of the epitopes that are listed in Table 1 in their bound conformations. These epitope molecules were built using ~150 residue backbone templates taken from epitope structures that were collected in SCEptRe (Table S4) and AutoPeptiDB (Table S5), and available in the PDB. 

Theoretically, six types of peptide-MHC structures were possible: (1) peptide-HLA (MHC I), (2) peptide-HLA (MHC II), (3) peptide-HLA-TCR (MHC I), (4) peptide-HLA-TCR (MHC II), (5) peptide-HLA-BCR (MHC I), and (6) peptide-HLA-BCR (MHC II). In this study, types 1, 2, and 3 were considered. We modeled the binding of the epitopes to different HLA proteins from MHC class I and II, and to HLA-TCR (MHC I). In the peptide-HLA-TCR type of binding, the docking scores were mostly higher (as compared to the binary peptide-HLA complexes). This was because epitope molecules were confined to the interface area between their cognate HLA/TCR proteins (Figure 4C). This mode of binding implies that that N- and C-termini are bound to the HLA surface, whereas middle residues interact with TCR.

Using the crystal structure of the nonapeptide **KTFPPTEPK** bound to HLA-A*1101 (PDB ID 1x7q) as the reference state, we performed an extensive conformational sampling and docking study of this complex. We demonstrated that the top-score docking peptide conformations were clustered around the native conformation, with an estimated energy −9.97 kcal/mol (corresponding to the nanomolar affinity range). Moreover, we found similar binding energies (~−9.5 kcal/mol) in docking simulations of **KTFPPTEPK** binding with HLA-A*02:01 (epitopes from Table 1, Table S6). Therefore, the computational protocol we used (see Methods) enabled: (1) the generation of a library of immunogenic sequences, and (2) structure-based selection of appropriate candidates using docking to multiple HLA structural templates. This approach was applied to all of the epitopes listed in Table 1. Some of these immunogenic sequences constitute overlapping sites. For example, the sequence of the reference nonapeptide (**KTFPPTEPK**) was identical to region Lys362–Lys370 in the SARS-CoV nucleocapsid protein. In the SARS-CoV-2 variant, this motif was predicted in the epitope sequences LNKHIDAY**KTFPPTEPK**, KHIDAY**KTFPPTEPK**KDKKK, and Y**KTFPPTEPK**KDKKKK, corresponding to positions Lys361 to Lys369 (Figure 4A, sky-blue area on the nucleocapsid protein surface). The nonapeptide **KTFPPTEPK** has demonstrated high-affinity binding to the protein from MHC Class I, whereas its interaction with the HLA-DRB1 (from MHC Class II) is less pronounced (estimated binding energy is ~−6–7 kcal/mol). Vice versa, extended peptides LNKHIDAY**KTFPPTEPK** (length 17), KHIDAY**KTFPPTEPK**KDKKK (length 20), and Y**KTFPPTEPK**KDKKKK (length 16) do not fit HLA binding sites in HLAs from MHC Class I. Interestingly, we found that the core part (**KTFPPTEPK)** of the LNKHIDAY**KTFPPTEPK** peptide can bind to the recognition site of HLA proteins from MHC Class I (~-7–8 kcal/mol), whereas the N-terminal part of this 17-residue peptide is arranged outside the A-pocket. The C-terminal part was found to occupy the F-pocket of the binding site (Figure 4D). In agreement with the well-known binding mode in the peptide-MHC class II system, the 17-residue peptide LNKHIDAY**KTFPPTEPK** demonstrated high-affinity docking scores, ~−9–10 kcal/mol, in interaction with DRB1 proteins. Accordingly, our molecular docking studies imply that peptides consisting of 9–11 amino acids were mostly recognized by MHC Class I molecules, whereas longer sequences tend to target the MHC Class II system (Figure S1). We predicted the MHC-I processing of identified immunodominant epitopes (Table 1) for all of the available MHC alleles of HLA-A, HLA-B, and HLA-Cusing the IEDB tool (http://tools.iedb.org/processing/) [20], and found that all of the immunodominant epitopes can undergo further proteolysis and recognition by MHC class I molecules (considering a processing score >1). Therefore, the core part of immunodominant epitopes with longer sequence lengths can be presented by MHC class I molecules after proteasomal processing.

## 4. Discussion

Vaccination is an effective way to improve public health by building up adaptive immunity to a target pathogen [52]. However, it takes considerable time to screen vaccine targets for clinical validation and the production of a vaccine. Advances in bioinformatics and next-generation sequencing technology, immunoinformatics, and reverse vaccinology can minimize the time for screening antigens from protein sequences of pathogens and offer advantages in the search for potential new vaccine targets [53,54]. Several antiviral drugs have been tested against COVID-19, however, none of these drugs proved to be completely effective against the disease [55]. The current global emergency of the COVID-19 outbreak urgently calls for a vaccine against SARS-CoV-2 [56]. Therefore, identifying which part of the sequence of SARS-CoV-2 proteins that can generate an immune response in humans will facilitate designing a vaccine against this viral pathogen [57]. While a few genetic variations exist between SARS-CoV and SARS-CoV-2, these viruses are more than 85% identical in their genomic sequences [58]. When considering the high genetic similarity between SARS-CoV and SARS-CoV-2, a few recent studies identified all of the completely identical B-cell and T-cell epitopes from the SARS-CoV-2, based on the experimentally-determined SARS-CoV epitopes that are present in the IEDB [47,59]. However, knowledge is still lacking before a full picture on SARS-CoV-2 epitopes that could have immunomodulatory effects in humans can be presented [60].

In this present study, we exploited immunoinformatics-based approaches to identify potential immunodominant epitopes from SARS-CoV-2, which could be useful for developing vaccines for the COVID-19 disease. The vaccines should be capable of activating both humoral and cellular immune responses in humans. Our approach to defining immunodominant epitopes entails identification of overlapping regions of B-cell and T-cell epitopes (MHC-I and MHC-II) from proteins of SARS-CoV-2, particularly at those sites where these epitopes are 100% identical to the experimentally-validated epitopes of SARS-CoV. We identified 15 potential immunogenic regions from three proteins of SARS-CoV-2, and mapped 25 epitopes that are 100% identical to experimentally validated SARS-CoV epitopes. Among 25 potential immunodominant epitopes identified containing 9–28 amino acid residues, the lengths of most of the epitopes were 16–18 residues. To understand the binding patterns of epitopes with MHC-I and MHC-II, we performed structural and molecular docking analyses. We found that in the library of our immunogenic sequences, epitopes 9–11 residues in length were mostly recognized by HLA proteins from MHC Class I, whereas longer epitopes tended to bind to MHC Class II proteins with higher affinities. This finding is in agreement with known canonical preferences. Further analysis of MHC class I processing reveals that epitopes of longer sequences can undergo proteasomal processing and that the core part of the region for MHC class I recognition within the epitope can be presented on the cell surface for surveillance by CD8 T-cells. An analysis of the population coverage by HLAs revealed seven epitopes among the predicted 25 immunodominant epitopes that are found in more than 87% of the global infected population, and show high binding affinity to MHC-I and MHC-II, as evidenced from structural and docking analysis. Furthermore, these seven epitopes were predicted as being non-allergen, non-toxic, and of low risk of triggering autoimmune responses, which highlights their potential as successful vaccine targets. The viral epitopes that are least likely to mutate should be selected in order to develop an effective vaccine. Thus, we analyzed available SARS-CoV-2 genomes from various geographic locations to identify the percentage of mutations in suggested epitope regions. We found evidence of point mutations in a few epitopes of SARS-CoV-2 isolates from the USA. This suggests that human immune pressure-induced genetic drift plays a central role in the genetic adaptation of SARS-CoV-2. Interestingly, we did not find any point mutations in the mentioned seven potentially immunodominant epitopes. This result indicates that these seven epitopes are potentially effective vaccine candidates. Hence, the development of vaccines using these seven immunodominant epitopes could activate both humoral and cellular immune responses in humans, and that these epitopes could cover almost all of the worldwide population. Our results thus offer important insight for the development of a peptide vaccine for COVID-19. 

## 5. Conclusions

The COVID-19 outbreak is an emerging threat across the globe. Despite this, there are currently no permanent antiviral drugs or vaccine reported for fighting this disease. In the present study, we identified immunodominant epitopes from SARS-CoV-2 proteins that could induce both humoral and cell-mediated immune responses in humans, using the most comprehensive immunoinformatics approaches. Molecular docking of the immunodominant epitopes with HLA alleles supports their higher binding affinities within different HLA alleles. Further, seven potential immunodominant epitopes were shortlisted based on their higher conservancy, higher global population coverage, and significant interaction to MHC class I and class II alleles with high affinity. These epitopes have a low risk of being allergen, toxic, or generating autoimmune reactions. These finding highlight that these seven immunodominant epitopes could be the potential vaccine targets against SARS-CoV-2. The computational approaches that were used in this study could be a benchmark for the identification of immunodominant epitopes from other emerging pathogens, particularly, coronaviruses, in order to develop potential universal vaccines against various new strains.

## Figures and Tables

**Table 1:** Potential immunodominant regions of SARS-CoV-2 and the mapped epitopes in those regions.

Potential Immunogenic Regions from Proteins of SARS-CoV-2, Isolated in Wuhan-Hu-1 (NC_045512.2)	The Number of Epitopes Mapped	Potential Immunodominant Epitopes
Membrane glycoprotein (61–70)	1	TLACFVLAAV
Membrane glycoprotein (157–187)	3	GRCDIKDLPKEITVATSR PKEITVATSRTLSYYKL TSRTLSYYKLGASQRV
Nucleocapsid phosphoprotein (176–191)	1	SRGGSQASSRSSSRSR
Nucleocapsid phosphoprotein (240–264)	2	QQQGQTVTKKSAAEASKK KKSAAEASKKPRQKRTA
Nucleocapsid phosphoprotein (268–286)	1	YNVTQAFGRRGPEQTQGNF
Nucleocapsid phosphoprotein (292–330)	3	IRQGTDYKHWPQIAQFA QFAPSASAFFGMSRIGM FFGMSRIGMEVTPSGTW
Nucleocapsid phosphoprotein (360–375)	1	YKTFPPTEPKKDKKKK
Spike glycoprotein (327–343)	1	VRFPNITNLCPFGEVFN
Spike glycoprotein (663–680)	1	DIPIGAGICASYHTVSLL
Spike glycoprotein (817–833)	1	FIEDLLFNKVTLADAGF
Spike glycoprotein (891–918)	3	GAALQIPFAMQMAYRFN PFAMQMAYRFNGIGVTQ MAYRFNGIGVTQNVLYE
Spike glycoprotein (1019–1041)	2	RASANLAATKMSECVLG AATKMSECVLGQSKRVD
Spike glycoprotein (1060–1068)	1	VVFLHVTYV
Spike glycoprotein (1157–1209)	3	KNHTSPDVDLGDISGIN DLGDISGINASVVNIQK EIDRLNEVAKNLNESLIDLQELGKYEQY
Spike glycoprotein (1254–1273)	1	CKFDEDDSEPVLKGVKLHYT

**Table 2:** Epitopes with more than 85% world population coverage.

Epitopes	Epitope Location	World Population Coverage (%)	Predicted HLA Locus
PKEITVATSRTLSYYKL	Membrane glycoprotein: 165–181	95.82%	HLA-A, HLA-B, HLA-DRB1, HLA-DRB3, HLA-DRB4, HLA-DRB5, HLA-DQA1, HLA-DQB1
QFAPSASAFFGMSRIGM	Nucleocapsid phosphoprotein: 306–322	92.81%	HLA-A, HLA-B, HLA-DRB1, HLA-DRB5, HLA-DPA1, HLA-DPB1, HLA-DQA1, HLA-DQB1
FFGMSRIGMEVTPSGTW	Nucleocapsid phosphoprotein: 314–330	87.42%	HLA-A, HLA-B, HLA-DRB1, HLA-DRB4, HLA-DRB5, HLA-DQA1, HLA-DQB1
FIEDLLFNKVTLADAGF	Spike glycoprotein: 817–833	94.26%	HLA-A, HLA-B, HLA-DRB1, HLA-DRB3, HLA-DRB4, HLA-DRB5, HLA-DQA1, HLA-DQB1, HLA-DPA1, HLA-DPB1
GAALQIPFAMQMAYRFN	Spike glycoprotein: 891–907	97.46%	HLA-A, HLA-B, HLA-DRB1, HLA-DRB4, HLA-DRB5, HLA-DQA1, HLA-DQB1, HLA-DPA1, HLA-DPB1
PFAMQMAYRFNGIGVTQ	Spike glycoprotein: 897–913	92.52%	HLA-A, HLA-B, HLA-DRB1, HLA-DRB3, HLA-DRB4, HLA-DRB5, HLA-DQA1, HLA-DQB1, HLA-DPA1
EIDRLNEVAKNLNESLIDLQELGKYEQY	Spike glycoprotein:1182–1209	88.57%	HLA-A, HLA-B, HLA-DRB1, HLA-DRB3, HLA-DQA1, HLA-DQB1

## Supplementary Materials

The following are available online at https://www.mdpi.com/2076-393X/8/2/290/s1. Figure S1: Typical binding mode of an elongated epitope in the HLA protein from MHC class II. Table S1: Details of all the predicted immunodominant epitopes of SARS-CoV-2, Table S2: SASA values calculated for the epitopes presented in Figure 2D,E, Table S3: SASA values calculated for all residues of the SAR-CoV-2 Spike glycoprotein, Table S4: Epitopes retrieved from SCEptRe which was used as backbone templates for structural analysis, Table S5: Epitopes retrieved from AutoPeptiDB which were used as backbone templates for structural analysis, Table S6: Identification of the binding core part in potential immunogenic epitopes, and binding/docking energies in HLA proteins from MHC Class I and II.
